# Effect of a Nutritional Support System to Increase Survival and Reduce Mortality in Patients with COVID-19 in Stage III and Comorbidities: A Blinded Randomized Controlled Clinical Trial

**DOI:** 10.3390/ijerph19031172

**Published:** 2022-01-21

**Authors:** Fernando Leal-Martínez, Lorena Abarca-Bernal, Alejandra García-Pérez, Dinnaru González-Tolosa, Georgina Cruz-Cázares, Marco Montell-García, Antonio Ibarra

**Affiliations:** 1 Centro de Investigación en Ciencias de la Salud (CICSA), Facultad de Ciencias de la Salud (FCS), Universidad Anáhuac México Norte, Mexico City 52786, Mexico; lore19.ab@gmail.com (L.A.-B.); alejandragp_@hotmail.com (A.G.-P.); din.tolosa@gmail.com (D.G.-T.); georgina.crucaz@hotmail.com (G.C.-C.); jose.ibarra@anahuac.mx (A.I.); 2 Centro Médico ISSEMyM (CMI) Toluca “Lic. Arturo Montiel Rojas”, Toluca City 52170, Mexico; cazabichos@yahoo.com

**Keywords:** COVID-19, SARS-CoV-2, nutritional support, NSS, nutrition, supplementation, probiotics, pneumonia, survival, mortality

## Abstract

The COVID-19 evolution depends on immunological capacity. The global hospital mortality rate is 15–20%, but in México it is 46%. There are several therapeutic protocols, however, integral nutrition is not considered. In this study, a Nutritional Support System (NSS) was employed to increase survival and reduce mortality in patients with stage III COVID-19. A randomized, blinded, controlled clinical trial was performed. Eighty patients (aged 30 to 75 years, both sexes) were assigned to (1) “Control Group” (CG) hospital diet and medical treatment or (2) “Intervention Group” (IG) hospital diet, medical treatment, and the NSS (vitamins, minerals, fiber, omega-3, amino acids, B-complex, and probiotics). IG significantly increased survival and reduced mortality compared to CG (*p* = 0.027). IG decreased progression to Mechanical Ventilation Assistance (MVA) by 10%, reduced the intubation period by 15 days, and increased survival in intubated patients by 38% compared to CG. IG showed improvement compared to CG in decrease in supplemental oxygen (*p* = 0.014), the qSOFA test (*p* = 0.040), constipation (*p* = 0.014), the PHQ-9 test (*p* = 0.003), and in the follow-up, saturation with oxygen (*p* = 0.030). The NSS increases survival and decreases mortality in patients with stage III COVID-19.

## 1. Introduction

The SARS-CoV-2 disease was first identified in Wuhan, China, in December 2019 [1] and was declared a global pandemic on March 11, 2020, by the World Health Organization [2]. Epidemiologically, according to the WHO until December 2021, there are a total of 270,155,054 confirmed cases with a total of 5,305,991 deaths, with a 2.18% mortality rate and a hospital mortality rate between 15% and 20% [3]. In Mexico the number of deaths amounts to 296,984. The mortality rate is 9.07% [4,5] with hospital mortality of 46% and 75% among intubated patients [6,7]. Comorbidities such as obesity, systemic arterial hypertension (SAH), and type 2 diabetes mellitus (DM 2) have been associated with more severe disease symptoms [8]. Treatment is not specific; international guidelines recommend analgesia, retroviral, steroids, anticoagulation, and the occasional use of monoclonal antibodies, depending on the stage, the thrombotic risk, and renal and liver function [9].

Regarding nutritional support, the European Society for Parenteral and Enteral Nutrition (ESPEN) Guidelines recommend an energy calculation of 27–30 kcal/kg/day, proteins up to 1.5 g/kg/day, carbohydrates, and lipids with a proportion of 30:70 and 50:50 in patients with respiratory failure [10]. The evidence of nutrients, probiotics, minerals, and certain vitamins for therapeutic use is limited, although some are studied to improve the prognosis of patients [11]. It is known that patients with COVID-19 are catabolic and decrease their protein reserve together with cachexia and hyporexia, provoking nutritional deficit and dysbiosis [12,13]. If malnutrition prevails, it compromises immunity and inflammatory response, so a Nutritional Support System (NSS) may be necessary to reduce complications due to SARS-CoV-2, the progression to MVA, and even death [14]. The aim of this study was to investigate the effect of the NSS to increase survival and reduce mortality in patients with stage III COVID-19 and comorbidities. 

## 2. Materials and Methods

### 2.1. Subject Selection

Inclusion criteria: hospitalized COVID-19 patients aged 30 and 75 years of both sexes, with oxygen saturation < 90%, use of supplemental oxygen, tolerance to the oral route, comorbidities (DM 2, SAH, and Body Mass Index (BMI) 25–40) were interviewed. 

Non-inclusion criteria: respiratory frequency (BR) > 30 bpm, glycemia > 250 mg/dL, severe gastroesophageal reflux and/or dysphagia, a neurological condition, surgery < 3 months, allergy to any component of the NSS, sepsis, cancer, or chronic degenerative diseases that increase the metabolism.

### 2.2. Trial Design and Supervision

A randomized, blinded, controlled clinical trial was conducted at Centro Medico ISSEMyM Toluca (CMI), State of Mexico. The clinical phase started in September 2020. At discharge, each patient was followed for 40 days or until death. Recruitment ended in February 2021 and follow-up ended in April 2021.

Sampling and group assignment: Consecutive cases. Randomization was performed in blocks of 10 patients, assigned by a sequence of random numbers obtained from the Excel program and divided into two groups. 

A triple blinding was planned: 1. Participants had no contact with each other because they were isolated, and when collecting informed consent, they were made aware that nutritional support might or might not be included with their meals as well as being part of regular medication or not; 2. The evaluators (treating physicians, care, and laboratory personnel) were unaware of the patient’s allocation; 3. Outcomes Assessors.

**Figure 1:**
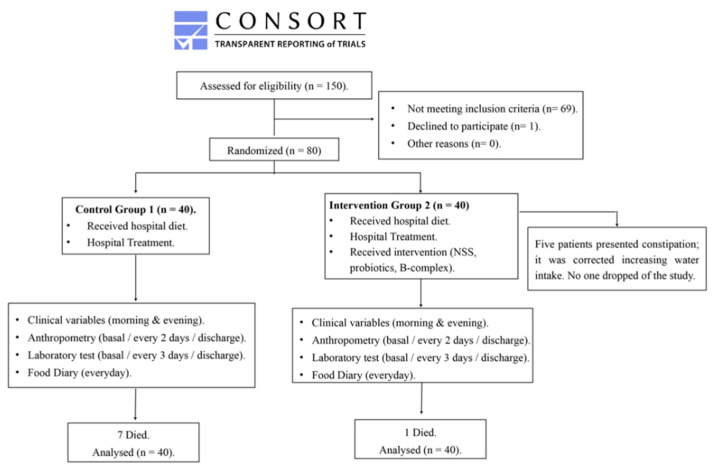
Consort. Transparent reporting trials.

One hundred and fifty patients were interviewed, 80 were chosen and randomized, and 40 patients were assigned to each group. The two groups were: 1. Control Group (CG); and 2. Intervention Group (IG) (Figure 1).

After deaths, there were 33 patients in the CG and 39 patients in the IG who completed the follow-up on day 40. The nutritional adequacy and calculation of all the patients in the study were performed by the hospital nutrition department before they were enrolled in the study; this department did not know the assignment of the patients. The nutritional intake in the baseline was assessed by food diary analysis. Food diaries were applied and collected by the research team; each patient was followed up from the initial interview until hospital discharge.

The IG received the NSS, which consists in:

(1) B-complex: 10 mg of cyanocobalamin, 100 mg of thiamin, and 100 mg of pyridoxine administered intramuscularly every 24 h for the first 5 days.
(2) NSS powder: one envelope orally after morning meals and another after evening meals, diluted in 400 mL of water each, during the whole intervention for a maximum of 21 days. Each envelope contained: *Spirulina Maxima* 2.5 g, folic acid 5 mg, glutamine 5 g, vegetable protein 10 g, constituted by two foods without processing or fragmenting in amino acids, brewer’s yeast, and amaranth, ascorbic acid 1 g, zinc 20 mg, selenium 100 mcg, cholecalciferol 2000 IU, resveratrol 200 mg, Omega-3 fatty acids 1 g, L-Arginine 750 mg, inulin 20 g, and magnesium 400 mg.
(3) Probiotics: *Saccharomyces boulardii* (SB) 500 mg daily for 6 days orally.

Table 1 summarize the effects of the nutrients contained in the NSS.

The clinical staff, evaluators, and patients were unaware of the treatment assignments. The research team was responsible for the administration of the NSS without any other patient or treating physician in the study being aware of it. 

Discharge criteria were: spO2 > 90% with a minimum of 2 L of supplemental oxygen, BR < 20 bpm, T° < 37 °C, with no data of sepsis. 

### 2.3. Clinical and Laboratory Monitoring

Patients were evaluated before starting the study and during their hospital stay by physical examination, vital signs (spO2, HR, BR, BP, and T°), food diary, lipid profile, glucose, serum electrolytes, liver function tests, renal function, hematic biometry, procalcitonin, ferritin, CPR, fibrinogen, and D-dimer every third day. Clinical tests were applied such as: MNA, PHQ-9, Bristol scale, and q-SOFA. 

### 2.4. Statistical Analysis

Data distribution in numerical variables was analyzed using Shapiro–Wilk normality test. For the analysis of overall survival and mortality, progression to ventilation, and survival and mortality in intubated patients, Kaplan–Meier curves and significant difference by Log-Rank were used. 

For the intergroup comparison analysis of the numerical variables, a *t*-test or Mann–Whitney test was used. For the intragroup analysis, paired *t*-test or Wilcoxon test was used. 

For the analysis of qualitative variables, the Fisher’s exact test (one-sided) was used; *p* values ≤ 0.05 were considered indicative of statistical significance. The programs used were Prism 6 and SPSS Statistics version 25.

### 2.5. Sample Size 

The sample size was calculated using the following formula: *n* = $\frac{\left(z^{2}pq\right)}{e^{2}}$, where: confidence level 95% (*z* = 1.96), *p* = 0.975, *q* = 0.025, with a margin of error of 5% (*e* = 0.05), obtaining 38 patients per group.

### 2.6. Ethical Aspects

Informed consent was obtained from all participants. The Declaration of Helsinki, the Nuremberg Code, and the Mexican Standard NOM-012-SSA3-2012 were considered. The research was approved by the research committee of the Faculty of Health Sciences of the Universidad Anáhuac México Norte, registration number: 202062, CONBIOETICA-15-CEI-004-20160729, the research committee of the CMI Toluca, registration number: 207C0401010201S/JIC/155/2020, and Clinical Trials registration code: NCT04507867.

## 3. Results

Eighty patients were chosen and randomized, and 40 were assigned to each group. After the deaths, 33 CG patients and 39 IG patients completed follow-up at day 40. 

### 3.1. Baseline Results

Clinical, demographic, and biochemical characteristics were compared in both groups at baseline and the results are shown in Table 2A,B. No significant difference was found in any item.

Adherence to the treatment was strictly supervised by the research team, and more than 95% of the participants complied with the treatment. A food diary was kept for each patient, estimating their caloric intake. The average kcal consumed at the beginning of the study in the CG was 1411 kcal/day, and in the IG, 1376 kcal/day. On the third day the consumption in the CG was 1597 kcal/day, and in the IG, 1773 kcal/day, with a 23% difference in caloric intake in favor of the IG.

### 3.2. Survival and Mortality of the Studied Patients

**Figure 2:**
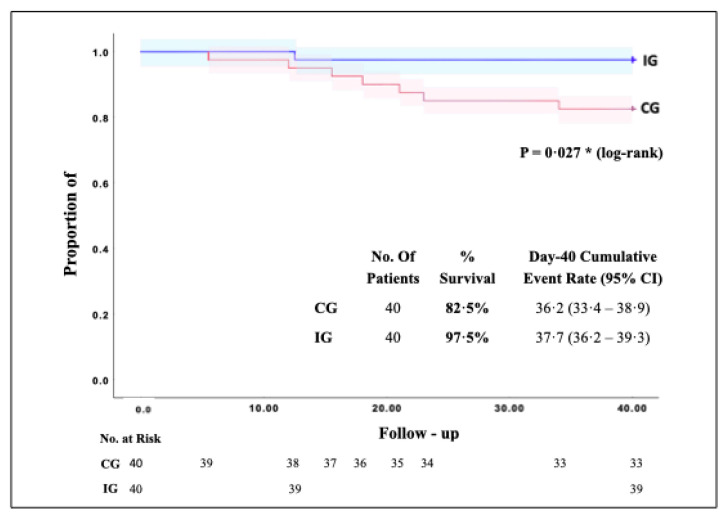
Kaplan–Meier general survival curve from CG and IG patients. The proportion of patients in the IG (blue line) and the CG (red line) included in the study and completed a 40-day follow-up from September to April 2021. The cumulative proportion of surviving patients was estimated with the Kaplan–Meier method and compared in both groups, from the CG died seven of 40 patients (surveillance 97.5%, mean 39.31%, 95% of (CI), 37.98 to 40.64). This showed a significant difference of IG compared to CG * p = 0.027 (Log Rank test, 95% of (CI).

Overall survival and mortality at day 40 are shown in Figure 2. Seven of 40 patients died in the CG (survival 82.5%, mortality 17.5%, median survival 36.22 days, 95% confidence interval (CI), 33.46 to 38.98 days), while one of 40 patients died in the IG (survival 97.5%, mortality 2.5%, median survival 39.31 days, 95% (CI), 37.98 to 40.64 days). In the IG (survival increased and mortality decreased compared to the CG) *p* = 0.027 (Log Rank). All the deceased patients were male. 

**Figure 3:**
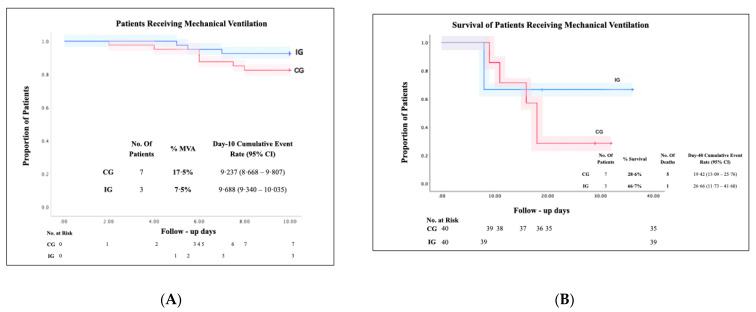
The proportion of patients with MVA from CG and the IG were included in the study and completed 40-day follow-up from September 2020 to April 2021. (**A**): IG and CG were analyzed with the Kaplan–Meier method comparing both groups to MVA progression, from CG seven patients progressed to MVA (17.5%, mean 9.68%, (CI) of 95%, 8.66 to 9.80); meanwhile, MVA progression of IG in comparison with CG. (**B**): In overall survival and mortality with MVA, five out of seven patients died in CG) 71.4%, mean 19.42, (CI) of 95%, 13.09 to 25.76). One out of three patients died in IG (33.5%, mean 26.66, (CI) of 95% 11.73 to 41.6), this represents the 38.1% decrease in mortality with MVA in IG compared to CG.

Of the patients who progressed to MVA, seven were from CG (17.5%) and three from IG (7.5%), representing a 10% decrease in IG compared to CG (Figure 3A). Survival in CG corresponded to two of seven patients (28.6%), while in IG two of three patients (66.7%) survived, increasing survival in IG by 38.1% compared to CG, and decreasing mortality to 33.3% of patients with MVA in IG compared to 71.4% in CG (Figure 3B).

In the analysis of the days with MVA of the patients who survived, the CG presented a mean of 26 days ± 2.83 while the IG presented 11 days ± 0, decreasing 15 days with MVA in favor of the IG.

The oxygen flow administered to maintain spO2 > 90% from baseline to day three was analyzed in both groups. In the CG a mean of 5.9 L (±3.8) was found in the baseline in 6 L (±4.4) at day three, while the IG presented a mean of 6 L (±3.2) in the baseline and 4.5 L (±3.5) at day three, which represents a significant difference in the decrease in oxygen flow in the IG (*p* = 0.014, Wilcoxon test). In addition, the presence of spO2 > 90% at day three was analyzed between both groups after oxygen supplementation. In the CG, 85% of the patients showed progress as did 92.5% in the IG, with a difference of 7.5% in favor of the IG. 

To evaluate prognosis, the qSOFA scale was applied and the baseline was analyzed and compared to day three in both groups. The CG presented a mean of 0.425 points (±0.594) at baseline and a mean of 0.512 points (±0.571) at day three, while the IG presented a mean of 0.65 points (±0.62 at baseline and 0.43 points (±0.496) at day three, representing an improvement in the prognosis of the IG (*p* = 0.040, Wilcoxon test). 

The gastrointestinal function was analyzed through average bowel movements, abdominal distention, and the Bristol scale, reflecting a clinical improvement in IG (Table 2A). 

The depressive state was assessed using the PHQ-9 questionnaire at baseline and hospital discharge. In the CG a mean of 3.66 points (±2.5) was obtained at baseline and 1.9 points (±1.4) at day three, presenting a significant difference in the improvement of mood in the IG patients (*p* = 0.003, Mann–Whitney test). The above results are shown in Table 3A. Table 3B shows laboratory data that were associated with overall mortality.

### 3.3. Follow-Up

Patients who survived had a follow-up on day 40. Analysis of spO2 without supplemental oxygen was performed. In the CG, a mean of 90.30% (±3.4) was presented while the IG presented a mean of 92.08% (±2.5), which implies greater progression in spO2 in favor of the IG (*p* = 0.03, Mann–Whitney test).

At discharge, 85.2% of the CG and 66.7% of the IG required the use of home oxygen use, the CG presented a mean of 57.6 days (±24.6), while the IG presented a mean of 43.8 days (±16.2), a 13.8-day average reduction favoring the IG (Table 3A).

Post-COVID syndrome was analyzed and present in 37.5% of the patients in the CG and 23.5% of the IG (Table 3A). The decrease in body weight was present in 72.2% of the patients in the CG and 44.4% in the IG (Table 3A).

**Figure 4:**
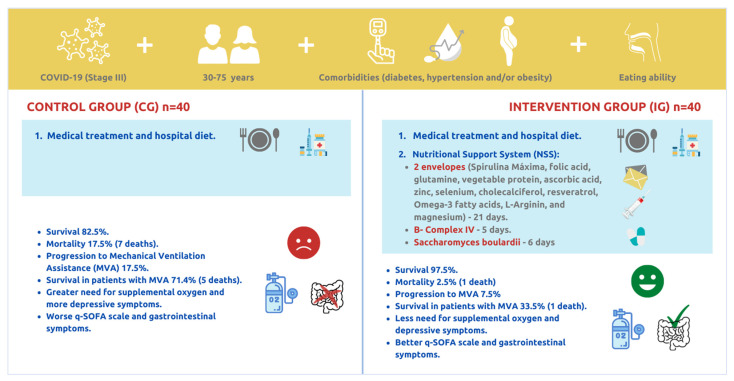
Characteristics of the study groups and primary outcomes.

The schematization of the study groups and the primary results are shown in Figure 4. 

## 4. Discussion

SARS-CoV-2 is a challenge for researchers due to current therapeutic limitations. Some drugs that have achieved positive results are Tocilizumab, reducing progression to MVA 12% [14], and Remdesivir, decreasing mortality 6.7% at the hospital level. The use of these two drugs in therapy, however, is limited by their side effects [45].

Altered nutritional status is known to be associated with decreased immunity and increased mortality in patients with COVID-19 [46]. It has been reported that decreased appetite and caloric intake are associated with increased synthesis of pro-inflammatory interleukins, mainly TNF-alpha, leading to cachexia [47]. Cachexia is associated with the reduction in glutamine and arginine reserves and the pool of amino acids, this generates alteration of the immune and ventilatory response in COVID-19 [21,48]. It is expected that providing nutritional support to these patients may improve their prognosis. 

The MATH+ Hospital Treatment Protocol for COVID-19 guidelines recommend the use of Vitamin D, Zinc, and melatonin, although their evidence on mortality and survival is limited [49]. It is known that the state of mind and the ability to sleep in patients with COVID-19 is associated with survival and mortality [50], it is also known that the consumption of probiotics, amino acids such as tryptophan and glutamine, and B complex, increase the production of neurotransmitters that regulate sleep and state of mind. [51] 

In this research, it was observed that NSS in the IG increased survival by 97.5% and reduced mortality by 2.5% compared to CG (*p* = 0.027). These results are significant, especially considering that hospital mortality worldwide in these patients is 15–20% [52]. The most evident results associated with clinical improvement were observed from day 3, a crucial moment to know the patient’s prognosis and which coincides with the increase in the cytokine storm [53]. At this point, spO2, abdominal distension, stool consistency, appetite, and water intake improved in the IG.

SARS-CoV-2 viral replication is directly associated with mortality [54]. The use of various nutrients in the regulation of different molecular pathways in viral processes has been demonstrated. Masuda et al. described that C-phycocyanin, an extract of Spirulina Maxima, blocks in vitro hemagglutination of viruses such as influenza, and the use of B complex and minerals such as zinc have been studied for their binding to the active site of the nsp 12 and 3C-like protein of SARS-CoV-2, blocking viral RNA transcription [55]. Folic acid (FA) inhibits the furin protease that SARS-CoV-2 needs to enter the host cell and its components (histidine and lepidine) block SARS-CoV-2 transcription and replication by forming hydrogen bonds with the SARS-CoV-2 enzyme 3CL hydrolase, counteracting 3CLpro (Mpro) which counteracts the host innate immune response [19]. It has been seen that when 200 µg/day of selenium is administered, it decreases SARS-CoV-2 transcription and replication, inhibiting the major protease (Mpro) [36].

It is known that dysbiosis is recurrent in SARS-CoV-2 [56] and that clinical improvement is associated with its correction. A microbiota–gut–lung axis has been described that coordinates inflammation and immunity mediated by T lymphocytes, it is dependent on eubiosis and the use of probiotics. *Saccharomyces boulardii* and *Saccharomyces cerevisiae* are one of the most studied probiotics due to their immunological effect [26,41]. These activate the Th1 andTh2 response, endocrine regulation, orexigenic factors, endothelial repair, and chemotaxis [37]. According to Youseff et al., the latter is also regulated by vitamin D, decreasing the expression of proinflammatory cytokines (IL-6) and the activation of TNF-alpha [57]. Zhang et al. point out that correcting dysbiosis decreases proinflammatory cytokines (IL-12, IL-6) and neutrophil infiltration in the lungs [58]. In addition, remote effects of this axis, mediated by Short Chain Fatty Acids (SCFA), are known [32,41,59]. One of the components of NSS is inulin. The positive effects of dietary fiber and inulin are known to promote the production of SCFAs by fermentation in the intestine and together with omega-3 PUFAs regulate TNF-alpha which is directly related to the inflammatory cascade and cachexia, two physiological processes related to mortality in patients with COVID-19 [27], especially in overweight, obese, and DM2 patients (Birkeland and Van der Beek) [60,61].

Amino acids such as glutamine and arginine have been used to increment the ventilatory response and they are currently being used to increase alveolar exchange in lung cancer and asthma [21,42] and are also necessary to produce collagen in pulmonary fibroblasts [23]. In SARS-CoV-2; specifically, the viral spike protein is responsible for the entry of SARS-CoV-2 into host cells and harbors arginine residues that modulate the binding of the receptor to the virus membrane [41,62]. Other nutrients such as selenium, and Omega-3 PUFAs participate in alveolar distention together with the surfactant factor [35] and Zinc that inhibits SARS-CoV-2 replication in vitro through inhibition of RNA-dependent RNA polymerase [32].

Patients who did not progress satisfactorily in the present study required MVA; upon admission to intensive care, catabolism, and oxidative stress increase, affecting prognosis; in Mexico, 75% of patients with COVID-19 and MVA die [7]. Related to this, it is known that arginine and glutamine interact in the homeostasis of reactive oxygen species (ROS) regulating oxidative stress. In recent studies, it was demonstrated that the use of 10 g of glutamine three times a day improves saturation and reduces progression to MVA and admission to the Intensive Care Unit (ICU) [47]. On the other hand, thiamine inhibits carbonic anhydrase isoenzymes in vitro and could limit hypoxia and decrease hospitalization in early stages of COVID-19 [63,64].

Progression among intubated patients to MVA was reduced to 10%, while mortality was reduced to 33% in this study. Associated with the result of the qSOFA where the prognosis favored the IG group. Associated with this, the progression of the patients at discharge was favorable for the IG, where the amount of home supplemental oxygen was reduced by 18.5% and 13.8 days less use in contrast to CG patients.

Folic acid, ascorbic acid, and resveratrol are associated with the survival and improvement of patients after infection, which regulate cell apoptosis induced by MERS-CoV-2 due to their anti-inflammatory and metabolic effects [19,32,40]. It is also known that the lymphopenia observed in most SARS-CoV-2 patients is associated with decreased levels of ascorbic acid and arginine. During SARS-CoV-2 infection, ascorbic acid modulates ROS production, decreases glyceraldehyde 3-phosphate dehydrogenase (GAPDH) enzyme activity, preventing cell death, regulation of lymphoid cell production [29] and pneumocyte preservation [32].

Improved mood and melatonin upregulation have been reported to be linked to prognosis in patients with COVID-19. When analyzing PHQ-9, there was a difference in favor of IG patients, which may be related to the gut–brain–microbiota axis [60]. The latter regulates human behavior and the circadian cycle through neurotransmitters such as serotonin, 95% of which is produced in the gut, and dopamine and melatonin, which are generated from microbiota and nutrients such as tryptophan, pyridoxine, nicotinic acid, and cobalamin [16]. It is known that the state of mind and the ability to sleep in patients with COVID-19 is associated with survival and mortality, it is also known that the consumption of probiotics, amino acids such as tryptophan and glutamine, and B complex, the increase the production of neurotransmitters that regulate sleep and state of mind [65]. 

## 5. Conclusions

Our study provides important results on the positive effect of NSS on survival and decreased mortality in these hospitalized patients with COVID-19, associating that the use of different nutrients together, in addition to the adequate regulation of the microbiota, is determinant for the clinical improvement, prognosis, and evolution of the disease.

Although more multicenter studies are needed to corroborate the impact of these findings and to replicate the results obtained to recommend their use, this study leaves a precedent of a significant effect in a small sample that may serve other researchers to consider complementing new therapeutic lines.

## Figures and Tables

**Table 1:** Biological functions of NSS components.

Specific Nutrients in the NSS	Dose	Function
Cyanocobalamin	10 mg	B complex has demonstrated effects on immune response, proinflammatory cytokine levels, respiratory function, endothelial integrity, and hypercoagulability. B complex binding to the active site of the nsp 12 and 3C-like protein of SARS-CoV-2, blocking viral RNA transcription [15]. Thiamine inhibits carbonic anhydrase isoenzymes in vitro and could limit hypoxia and decrease hospitalization in early stages of COVID-19 [16]. Pyridoxine upregulates IL-10. Cobalamin modulates gut microbiota and low levels elevate methylmalonic acid and homocysteine, increasing inflammation, reactive oxygen species, and oxidative stress. Methylcobalamin forms adenosylcobalamin for mitochondrial energy production [17].
Thiamin	100 mg	
Pyridoxine	100 mg	
*Spirulina Maxima*	5 g	*Spirulina Maxima* (SM) is a natural biological source of ACE inhibitory peptides, which are used to enhance ACE2 activity for the treatment of tissue injury of several organs in severe cases of coronavirus infection. ACE inhibitory peptides modulate oxidative stress, cytokine release syndrome, and tissue injury in SARS-CoV2 and other coronavirus infections through regulation of ACE2 activity [18]. C-phycocyanin, an extract of SM blocks in vitro hemagglutination of viruses such as influenza [15].
Folic acid	5 mg	Folic acid (FA) inhibits the furin protease that SARS-CoV-2 needs to enter the host cell; FA components (histidine and lepidine) block SARS-CoV-2 transcription and replication by forming hydrogen bonds with the SARS-CoV-2 enzyme 3CL hydrolase, counteracting 3CLpro (Mpro) which counteracts the host innate immune response [19]. FA inhibits RNA expression of various viruses at the post-transcriptional level [20].
Glutamine	10 g	Glutamine (Gln) stimulates the immune system by inhibiting inflammatory responses [21]. In COVID-19 the depletion of Gln reserves represses the increase in CD8+ T lymphocytes, IL-2 and IFN-γ [22]. Gln stimulates collagen production in fibroblasts and stimulates lung myofibroblast differentiation in patients infected with EBV, HVC, HV8, and SARS-CoV-2 [23]. Gln increases ventilatory response and is employed to maximize alveolar exchange in lung cancer and asthma [24]. Administering 30 g/day of Gln improves oxygen saturation and reduces progression to MVA, hospital stay, and intensive care unit (ICU) admission [25,26].
Vegetable protein (Brewer’s yeast-*Saccharomyces cerevisiae SC and Amaranth*)	20 g	The balance of proteins of high biological value (PHBV) increases antibody production, modulates inflammatory response, stimulates GALT, MALT, and BALT [27]. Brewer’s yeast provides *Saccharomyces cerevisiae* (SC) and has an immunomodulatory effect by stimulating GPCR/YSD, prevents ACE2 receptor binding to SARS-CoV-2 protein S, and modulates the activation of MAPKs, inhibiting the production of IL-1β, IL-6, and TNF-α [26]. SC in vitro promotes muscle amino acid reserve recovery during SARS-CoV-2 infection, and also regulates the Cop9 signalosome pathway (CSN), a protein complex that regulates Cullin-RING ubiquitin ligase (CRL) activity, thus preventing protein degradation [28].
Ascorbic acid	2 g	During SARS-CoV-2 infection, ascorbic acid (AA) modulates ROS production, reduces glyceraldehyde 3-phosphate dehydrogenase (GAPDH) enzyme activity, resulting in the prevention of cell death [29], pneumocytes preservation, and attenuates the activation of immune response [30,31]. AA inhibits immune cell lactate and preserves innate immunity of alveolar epithelial type II cells in SARS-CoV-2 infection [32].
Zinc	40 mg	Zinc (Zn) inhibits SARS-CoV-2 replication in vitro through inhibition of RNA-dependent RNA polymerase (RdRp) and 3C like viral proteinase (3CLpro). Zn upregulates TNF-α and IL-1β levels, modulates cytokine storm in COVID-19 [32]. In SARS-CoV-2 infection Zn enhances the synthesis of metalloenzymes, ACE and ACE 2, that when disabled have beneficial therapeutic effects on the disease progression [33].
Selenium	200 mcg	Selenium (Se) integrates glutathione peroxidases, selenoprotein F, K, S, and thioredoxin reductases (TXNRD), which promote differentiation and proliferation of innate and adaptive immunity [34]. Se participates in alveolar distention together with the surfactant factor. TXNRD1 upregulates Nrf2 in lung epithelial cells and decreases the expression of NF-κB, IL-1β, IL-6, and TNF-α, thereby regulating the immune and inflammatory response in COVID-19 [35]. Se increases the proliferation of T and NK lymphocytes; 200 µg/day decrease viral transcription and replication, inhibiting the major protease (Mpro) of SARS-CoV-2 [36].
Cholecalciferol	4000 IU	Cholecalciferol (D3) activates chemotaxis, decreasing the expression of IL-1, 2, 6, 12, IFN-γ, and TNF-α activation, inhibits antigen-presenting cells, therefore has anti-proliferative effect of T cells [37]. In COVID-19, D3 increases the proliferation and differentiation of keratinocytes, endothelial cells, osteoblasts, and lymphocytes; increases the production of IL-10, IL-4, IL-5, and transforms growth factor-β; D3 activates the TH1 response [38]. Its antioxidant effect increases Nrf2 factor activity, activates the Nrf2-Keap1 (Kelch-like ECH-associated protein 1) pathway and maintains redox balance in COVID-19. The effect on the regulation of RAAS (renin-angiotensin-aldosterone system), promotes the inhibition of renin gene transcription, blocking the CREB-CBP/p300 complex, which intervenes in the homeostasis of superoxide dismutase (SOD), Glucose 6-phosphate dehydrogenase, glutathione reductase (GR), and glutathione peroxidase (GP), inhibit genes that attenuate SAH, systemic inflammation, and renal and cardiovascular lesions [39].
Resveratrol	400 mg	Resveratrol inhibits NF-κB and activation of SIRT1 and p53 signaling pathways. Activation of SIRT1 increases NAD levels and enhances mitochondrial function, regulating the inflammatory response and dysfunctional physiological processes. The activation of SIRT1 and Superoxide Dismutase (SOD) by Resveratrol increased ACE2 function and decreased inflammation [40].
Omega-3 fatty acids	2 g	Omega-3 PUFA constitute membrane phospholipids and regulate membrane fluidity and protein complex assembly in lipid bilayers; modulate the expression, stability, and enzymatic activities of ACE2 and TMPRSS2. Omega-3 LC-PUFA, DHA regulates lipid raft formation [41]. ACE2 and TMPRSS2 are described to be present mainly in lipid pools. Lipid rafts have been shown to be involved in SARS-CoV entry into Vero E6 cells; 80% of the surfactant factor is constituted by phospholipids, mainly omega-3 PUFAs that contribute to adequate alveolar distension by regulating the production of type II pneumocytes and surfactant factor [32].
Arginine	1.5 g	Arginine (Arg) decreases cell apoptosis caused by SARS-CoV-2, it also matures CD3+ lymphocytes and regulates the production of CD8 T lymphocytes; When Arg reserves are decreased in COVID-19, it reduces the immune response through TLR4/MAPK signaling; in addition, the viral spike protein in SARS-CoV-2 contains arginine residues that modulate receptor binding to the virus membrane [42]. Arginine increases the ventilatory response and is used to increase alveolar exchange in lung cancer and asthma [23,24].
Magnesium.	800 mg	In SARS-CoV-2, magnesium (Mg) activates protein kinases, stimulates T-cell receptors and production by generating ATP, controls cell membrane inflammation, and has vasodilatory and antithrombotic effects. Is a modulator of the release of acetylcholine and histamine in the inflammatory cascade in viral infections [43]
*Saccharomyces boulardii* (SB)	500 mg	*Saccharomyces boulardii* (SB) has immunological effect, activates Th1 and Th2 response, improves endocrine regulation and increases chemotaxis. At intestinal level it significantly increases the concentration of IgA (important in SARS-CoV-2 diarrhea); also inhibits the synthesis of IL-8; reduces the activation of MAPK Erk1/2, JNK/SAPK and the nuclear translocation of NF-kB [44]. Increases short-chain fatty acids (SCFA) synthesis that regulate the immune response, inflammation and activates the gut-lung axis [41].
Inuline	20 g	Consumption of inulin-type fructans has been associated with regulation of the immune system, modulation of GI peptides, production of SCFA and modulation of triglyceride metabolism. SCFA are a source of energy for colonocytes, stimulating the synthesis of IL-10 by macrophages, neutrophils and T cells, as well as inhibiting the synthesis of TNF- α and IL-6 [27].

**Table 2:** (**A**) Demographic and clinical characteristics at baseline of CG and IG. (**B**) Clinical and biochemical characteristics at baseline of CG and IG.

(A)			
Characteristics	CG	IG	*p*-Value
	*n* = 40	*n* = 40	
Mean ± SD age—years	53.9 ± 10.3	51.5 ± 11.4	0.351
Female gender—no. (%)	13 (32.5)	15 (37.5)	0.407
Male gender—no. (%)	27 (67.5)	25 (62.5)	0.407
**Risk factors and coexisting conditions**—**no. (%)**			
Overweight	38 (95)	36 (90)	0.338
Obesity	14 (32)	13 (32.5)	1
DM 2	13 ± 0.325	11 ± 0.275	0.404
Cardiovascular disease	17 (42.5)	10 (25)	0.078
Hyperlipidemia	11 (27.5)	7 (17.5)	0.422
Gastrointestinal Disease	14 (35)	13 (32.5)	1
Total Risk Factors	2.92 ± 1.42	2.57 ± 1.35	0.261
**COVID-19 Symptoms**—**no. (%)**			
Dyspnea	24 (60)	26 (65)	0.409
Nausea and vomit	6 (15)	7 (17.5)	0.5
Hyposmia	12 (30)	15 (37.5)	0.637
Dysgeusia	18 (45)	20 (50)	0.412
Headache	26 (65)	29 (72.5)	0.315
Myalgia	32 (80)	30 (75)	0.395
Diarrhea	18 (45)	12 (30)	0.124
Anorexia	20 (50)	21 (52.5)	0.500
Total of symptoms	7.05 ± 2.11	6.8 ± 2.23	0.608
**Gastrointestinal Clinic**			
Bristol—no. (%)	9 (50%)	5 (27.8%)	0.153
No. of defecations—Mean ± SD	0.54 ± 0.6	0.52 ± 0.73	0.717
Abdominal distension—no. (%)	28 (70%)	28 (70%)	0.596
**Vital Signs Mean ± SD**			
Breathing Frequency—bpm	21.18 ± 3.01	21.48 ± 3.01	0.378
Oxygen Saturation—%	92.73 ± 4.17	94 ± 3.18	0.144
Heart Rate—bpm	70.7 ± 15.4	75.5 ± 9.88	0.105
Temperature—°C	36.27 ± 0.73	36.3 ± 0.62	0.935
Oxygen flow—L/min	5.9 ± 3.82	6 ± 3.29	0.571
Qsofa—pts	0.425 ± 0.59	0.65 ± 0.62	0.100
(**B**)			
**Characteristics**	**CG**	**IG**	***p*-Value**
	***n* = 40**	***n* = 40**	
**Nutritional Status**—**Mean ± SD**			
MNA^®^—pts	11.13 ± 2.26	11.38 ± 1.65	0.828
BMI—kg/m^2^	29.35 ± 3.89	29.98 ± 4.07	0.403
Hydric balance—mL/day	−203.4 ± 966	−301.5 ± 1167	0.806
**Medication**—**no. (%)**			
Antihypertensive	13 (32.5)	9 (22.5)	0.227
Antidiabetics	9 (22.5)	11 (27.5)	0.398
Antilipids	3 (7.5)	3 (7.50)	0.662
Antibiotics	1 (2.50)	4 (10)	0.359
Antiacids	6 (15)	6 (15)	0.662
NSAIDs	8 (20)	12 (30)	0.220
**Laboratory studies**—**Mean ± SD**			
Hb—g/dL	15.53 ± 2.222	15.54 ± 2.088	0.987
MCHC—G/Dl	33.39 ± 1.4	33.46 ± 1.17	0.753
Platelets—10^3^/µL	222.2 ± 53.93	248.4 ± 139.9	0.790
Leukocytes—10^3^/µL	8.97 ± 4.15	8.46 ± 4.36	0.400
Neutrophils—%	83.5 ± 8.87	80.78 ± 9.29	0.172
Glycemia—mg/dL	135.4 ± 59.39	134.8 ± 58.83	0.872
Total cholesterol—mg/dL	142.8 ± 42.82	135 ± 23.53	0.335
Triglycerides—mg/dL	147.5 ± 58.92	132.8 ± 37.39	0.533
AST—U/L	48.06 ± 28.81	46.4 ± 49.65	0.214
ALT- U/L	47.69 ± 31.9	50.44 ± 50.88	0.914
Albumin—g/dL	3.53 ± 0.44	3.57 ± 0.41	0.768
Ferritin—ng/mL	1070 ± 899.3	1270 ± 1142	0.572
Fibrinogen—mg/dL	592.2 ± 170.4	607.4 ± 162.9	0.721
CRP—mg/dL	157.3 ± 106.7	135.3 ± 94.92	0.313
D dimer—ng/dL	291.2 ± 179.9	444.9 ± 954.9	0.927
Creatinine—mg/dL	0.88 ± 0.31	0.86 ± 0.22	0.734
Urea—mg/dL	33.95 ± 15.84	32.95 ± 10.78	0.710
BUN—mg/dL	15.87 ± 7.39	15.43 ± 5.02	0.690
GFR—mL/min/1.73 m^2^	90.15 ± 20.2	93.59 ± 17.4	0.673
Procalcitonin—ng/mL	0.364 ± 0.6	0.18 ± 0.188	0.356

SD, Standard Deviation. CG, Control Group. IG, Intervention Group. The comparison of both groups in the baseline did not show a significant difference in any item analyzed in this study. SD, Standard Deviation. CG, Control Group. IG, Intervention Group. MNA, Mini Nutritional Assessment.

**Table 3:** (**A**) Clinical evolution and 40-day follow-up. (**B**) Association of baseline laboratory parameters and general mortality.

**(A)**									
	**CG**				**IG**				**Intergroup *p*-Value**
**Clinical Evolution**	***n***	**Baseline**	**Day 3**	***p*-Value**	***n***	**Baseline**	**Day 3**	***p*-Value**	
Oxygen flow—L (intragroup)	40	5.9 ± 3.8	6 ± 4.4	0.919	40	6 ± 3.2	4.5 ± 3.5	0.014 *	
qSOFA—pts	40	0.42 ± 0.59	0.51 ± 0.57	0.608	40	0.65 ± 0.62	0.43 ± 0.49	0.040 *	
Number of defecations on day 3	37		0.81 ± 0.90		36		1.41 ± 1.13		0.014 *
Distension on day 3	31		51.60%		31		19.40%		0.008 *
PHQ-9 test—pts (intragroup)	6	3.66 ± 2.5	1.50 ± 2.8	0.187	10	5.3 ± 3.4	1.9 ± 1.4	0.003 *	
Oxygen Saturation > 90% on day 3	40		85%		40		92.50%		0.241
Hydric Balance on day 3—mL	17		123.4 ± 453.8		18		456.6 ± 485.5		0.043 *
Bristol scale on day 3	24		33.30%		31		41.90%		0.356
**40-day follow-up**	***n***		**Day 40**		***n***		**Day 40**		
Saturation without Supplementary Oxygen—%	28		90.39 ± 3.4		38		92.08 ± 2.5		0.030 *
Need for home Oxygen flow—%	27		85.2%		39		66.70%		0.078
Time of home Oxygen use—days	17		57.6 ± 24.6		23		43.8 ± 16.2%		0.098
Post-COVID syndrome—%	24		37.50%		34		23.50%		0.195
Weight decrease—% of patients	11		72.70%		18		44.40%		0.135
Gastrointestinal symptoms—%	24		16.70%		37		8.10%		0.266
(**B**)									
	**Discharge (*n* = 72)**				**Death (*n* = 8)**				***p*-Value**
**Baseline Values**	***n***		**%**		***n***		**%**		
Fibrinogen > 700 mg/dL	13		18.50		6		75.00		0.002 *
Procalcitonin > 0.5 ng/mL	4		5.5		4		50		0.003 *
Blood Urea Nitrogen > 22 mg/dL	5		6.90		4		50		0.004 *
RCP > 150 mg/L	28		38.8		7		87.5		0.011 *
Neutrophils > 80%	45		62.50		8		100		0.031 *
Leukocytes > 10 × 10^3^/μL	18		25.00		5		62.50		0.040 *
Urea > 40 mg/dL	12		16.6		4		50		0.047 *

The following table shows the results of the intergroup analysis between the IG (Intervention Group) and the CG (Control Group) for different parameters in the baseline or with a complete 40-day follow-up. Deceased patients were excluded * *p*-value < 0.05. The following results show the association between laboratory parameters taken in the baseline, with the overall mortality. Analysis was performed with Fisher’s exact test.

## Data Availability

Data supporting the reported results can be obtained by contacting the corresponding author.
